# Application of Artificial Intelligence in the Management of Drinking Water: A Narrative Review

**DOI:** 10.7759/cureus.49344

**Published:** 2023-11-24

**Authors:** Revathi G Maroju, Sonali G Choudhari, Mohammed Kamran Shaikh, Sonali K Borkar, Harshal Mendhe

**Affiliations:** 1 Department of Community Medicine, Datta Meghe Medical College, Datta Meghe Institute of Higher Education and Research (DU), Nagpur, IND; 2 Department of Community Medicine, Jawaharlal Nehru Medical College, Datta Meghe Institute of Higher Education and Research (DU), Wardha, IND

**Keywords:** machine learning, artificial intelligence, water management, safe water, waterborne diseases

## Abstract

Waterborne illnesses are a significant concern worldwide. The management of water resources can be facilitated by artificial intelligence (AI) with the help of data analytics, regression models, and algorithms. Achieving the Sustainable Development Goals (SDGs) of the 2030 Agenda for Sustainable Development of the United Nations depends on understanding, communicating, and measuring the value of water and incorporating it into decision-making. Various barriers are used from the source to the consumer to prevent microbiological contamination of drinking water sources or reduce contamination to levels safe for human health. Infrastructure development and capacity-building policies should be integrated with guidelines on applying AI to problems relating to water to ensure good development outcomes. Communities can live healthily with such technology if they can provide clean, economical, and sustainable water to the ecosystem as a whole. Quick and accurate identification of waterborne pathogens in drinking and recreational water sources is essential for treating and controlling the spread of water-related diseases, especially in resource-constrained situations. To ensure successful development outcomes, policies on infrastructure development and capacity building should be combined with those on applying AI to water-related problems. The primary focus of this study is the use of AI in managing drinking water and preventing waterborne illness.

## Introduction and background

Water is the natural elixir of life, as the saying goes. Life must have access to clean water to survive. Nonetheless, we discover that waterborne infections cause devastation in sizable areas of developing and undeveloped countries. According to the WHO, 3.6 million people worldwide die from waterborne illnesses, with children accounting for around 2.2 million fatalities. Waterborne diseases are illnesses brought on by drinking water infected with pathogens like dangerous bacteria, viruses, protozoa, etc [1]. These harmful microorganisms contaminate water due to improper sanitation practices, and sewage spills into drinking water sources. Both governmental and non-governmental organizations (NGOs) have made every effort to raise the drinking water standard. But for most people, access to safe drinking water remains a pipe dream. To successfully address this worldwide threat, optimal solutions built on cutting-edge deep learning and machine learning developments can be deployed [2]. 

The leading causes of water pollution are bacteria, viruses, parasites, insecticides, pharmaceuticals, plastics, feces, radioactive materials, fertilizers, and pesticides. These compounds are frequently invisible contaminants since they do not permanently alter the color of water [3]. The bacteria known as Anthrax bacilli, found in tanning wastes, are the most dangerous water contaminants. They all cause water contamination, make it hazardous to drink, and if it is utilized, cause illnesses transmitted through water [4]. Waterborne infections are a big concern on a global scale. Around three million people globally die from water-related diseases, with 1.2 million fatalities occurring in children, according to a United Nations report [1].

Clean water is a vital component of our health. The WHO estimates drinking contaminated water can result in cholera, dysentery, typhoid, polio infections, and 485,000 annual diarrheal fatalities. Identifying pathogens is consequently crucial for treating and preventing aquatic illnesses. A ground-breaking development in object detection has been made possible by the advent of machine learning and deep learning, both powered by AI [2]. The current state of water resources makes it clear that better management is required. Recognizing, assessing, and elaborating on the value of water and incorporating it into decision-making is crucial for managing water resources sustainably and reasonably and achieving the Sustainable Development Goals (SDGs) of the 2030 Agenda for Sustainable Development of the United Nations [5]. An essential prerequisite for our survival is adequate water quality [6]. The likelihood of an outbreak of waterborne disease is increased in disaster-affected areas if clean water supplies are destroyed or combined with contaminated water. A nation like Haiti, where a sizable portion of the population lacks access to fresh water and even the most basic sanitation services, would suffer more in the aftermath of a disaster. Humanitarian relief and managing epidemic diseases would benefit significantly from early warning of waterborne diseases like cholera. Finding the appropriate characteristics to predict a future epidemic better is not easy in disease forecasting [7].

Methodology 

Literature searches for articles published between 2000 and 2023 were made using Google Scholar, Pub Med, and other databases. The terms "safe water," "waterborne diseases," "machine learning," and "future" were also utilized. The inclusion criteria were publications between 2000 and 2023, English-language publications, and articles discussing using AI in all situations to manage waterborne infections and drinking water. We excluded duplicates, abstracts, works written in languages other than English, unpublished works, and materials that didn't have much to do with AI and the management of clean, safe drinking water.

## Review

Need for clean and safe drinking water

A global requirement for clean water, sanitary conditions, and robust aquatic ecosystems is the treatment of water and wastewater [8, 9]. Furthermore, having access to high-quality water is necessary for having a sustainable economy [10]. The health effects of natural disasters are one of their most frequent side effects, and they are directly tied to the water and sanitation issues that develop after such events [11]. The physical health of displaced persons is at risk both during and after crisis scenarios because they have fewer opportunities to practice good personal hygiene and have enough access to fresh water. As a result, the likelihood of an increase in waterborne diseases in a post-disaster situation is higher due to inadequate water, sanitation, and hygiene (WASH) conditions [12]. Some of the most common waterborne pathogens and diseases caused by them are given below.

Bacteria like Escherichia coli, Yersinia enterocolitica, Leptospira spp., Campylobacter jejuni, Salmonella spp., Vibrio cholerae, Salmonella typhi, and Shigella spp. cause a variety of water-borne illnesses, from septicemia and gastroenteritis to specific ailments like yersiniosis, leptospirosis, reactive arthritis, salmonellosis, cholera, typhoid fever, and bacillary dysentery. Meningitis, infectious hepatitis, gastroenteritis, heart defects, and hepatitis A and E are caused by viruses such as rotavirus, coronaviruses, and enteroviruses. Diseases like amebiasis, cryptosporidiosis, and giardiasis, which cause fevers and diarrhea, are caused by protozoa, such as Entamoeba histolytica, Cryptosporidium, Microsporidia, and Giardia lamblia. Helminths like Necator americanus, Taenia spp., Enterobius vermicularis, Ascaris lumbricoides, and Ancylostoma spp. can cause a variety of worm-related waterborne diseases, including necatoriasis, taeniasis, enterobiasis, ascariasis, and ancylostomiasis. Effective prevention and treatment techniques are essential for public health programs, and understanding these organisms and the diseases they are associated with is imperative [13].

Natural disasters frequently result in food insecurity and malnutrition due to the destruction of agricultural regions and crops, which raises the risk of cholera and other outbreaks of diarrheal diseases [14]. The primary means of transmission of cholera is through the intake of water and food contaminated with Vibrio cholera, the disease's causative agent. The condition is common in places with poor sanitary and water infrastructure [15,16]. Over 100,000-150,000 people worldwide die from cholera each year [17,18]. To lessen consumers' risk from contaminated water, automatic anomaly detection monitoring is crucial in water utilities' distribution systems. Two significant issues and occurrences in the water quality anomaly identification domain are unbalanced class distribution and missing data. An overestimated classification accuracy can be produced by learning algorithms in an unbalanced dataset due to a bias favoring the majority class at the expense of the minority class. The effectiveness of learning algorithms in real-world water quality anomaly detection situations is significantly hampered by these two issues. So, in order to improve performance, they must be properly studied and handled.

AI

The aim of AI is to develop methods and equations that, when included with a system, allow the system to handle problems in a manner that is similar to a human [19]. AI is the ability of a system to carry out a set of tasks in a manner that is similar to how humans carry them out [20,21]. Any intelligent system needs to be able to learn in order to acquire new skills through experiments, store knowledge, and apply knowledge to solve issues. The ability of an AI system to learn, adapt, and gradually forget outdated or irrelevant information to enhance future scenarios is its key advantage. The foundation of AI is its ability to learn and its purported robustness. Because of this, we think there aren't any practical alternatives to AI techniques for creating autonomous systems that will help people. The main contribution of AI is to reduce the need for human participation and assistance in routine control and adaptability operations. Human intervention in artificially intelligent systems should become less active and humans should have more of an observer role [22].

According to an IBM (International Business Machines Corporation) business policy document [23], all facets and technologies of our lives are developing toward making a "smart planet." The goal of developing the field of AI is to create autonomous, intelligent systems. ChatGPT is one of the most sophisticated AI systems as of 2023; it is a component of strong big language models, specifically a sizable neural network trained on zetta-bytes of internet-based text data. It can comprehend spoken language and produce responses that resemble those of humans. Just to clarify, ChatGPT is only one illustration of the cutting-edge AI systems that are now in use. DeepMind, an Alphabet Inc. subsidiary, created an AI called AlphaGo. In 2016, AlphaGo made headlines after defeating Lee Sedol, the most intelligent Go player in the world, in a five-game match. An old Chinese board game, AlphaGo, has straightforward rules but is exceedingly difficult to play without human intuition. According to DeepMind researchers, their AI is all-purpose, which means it can do much more than just play Go. Engineers utilized it to address various issues, such as protein folding and controlling Google data centers' cooling systems. IBM created the AI system known as Watson. It was initially created as a question-answering chatbot based on neural networks and advanced natural language processing.

Additionally, Watson excels in healthcare applications since it can foretell the likelihood of skin malignancies based solely on a person's photograph. It can more accurately identify a variety of ailments like cancer, cardiovascular disease, heart disease, etc., and also makes drug recommendations. Several hospitals and health facilities throughout the world make use of Watson's skills [24].

The application of AI is widespread now. Accenture and Frontier Economics' investigation also shows that the technology has much more potential. According to the analysis, due to artificial intelligence, industrialized countries' productivity growth will improve by up to 40% by 2035 [25].

Role of artificial intelligence in safe water supply

A discipline of computer science that deals with the simulation of intelligent behavior in computers or a machine's ability to resemble intelligent human behavior is known as AI [26]. AI or machine learning is primarily used to make decisions about providing efficient water supply. These tasks include maximizing the information and data water utilities have access to improve service delivery, capital investment optimization, and operating cost reduction, including social and environmental externalities. Water utilities frequently adopt business practices from other industries, particularly those in the energy industry, without fully comprehending the underlying presumptions and repercussions of doing so [27]. Coastal distribution and seasonal climate dynamics have been connected in earlier research to the pathogenic Vibrio cholera bacteria that cause human cholera illness. Numerous possibilities exist for developing cholera-risk apps for the environment that use remote-sensed significant climate parameters and random forest classifiers.

More research on the present random forest model and its primary climate variables is based on cholera surveillance datasets in additional coastal areas affected by the outbreak to determine the method's relevance and effectiveness for cholera forecasting systems. Numerous significant outbreaks have been caused by decreasing water quality or failing drinking water infrastructure. These problems can be detected beforehand by a real-time drinking water quality monitoring system, which can then notify operators and prompt them to take the necessary action. Although often used for this purpose, Supervisory Control and Data Acquisition (SCADA) has several disadvantages, such as issues with sensor scalability, a lack of predictive capability, and increased effort for operators owing to the constant onslaught of pointless alarms. AI can facilitate the management of water resources with data analytics, regression models, and algorithms. These cutting-edge technologies make it easier to design efficient water networks and systems. AI makes it feasible to construct water facilities and assess the quality of the water supply. Governmental agencies and water managers can employ artificial intelligence to create an intelligent water infrastructure to manage water efficiently and adapt to the environment. These environmentally friendly and economically advantageous technologies will be able to fully use all available water management alternatives and foresee possible risks [28].

Over the world, significant outbreaks caused by water infrastructure failure or water quality degradation have often occurred. These problems can be detected beforehand by a real-time drinking water quality monitoring system, which can also notify operators to take the necessary action. Despite being commonly used for this purpose, SCADA has many limitations, including sensor scalability challenges, a lack of predictive capability, and an increased burden for operators bombarded with unneeded warnings. Cloud Internet of Things (IoT), AI and Soft Computing (AI and SC), and other technologies can reduce operator dependency and improve system operations [29].

Water contamination is the fundamental cause of many diseases in the world. Sensors must be employed to gauge the water's quality to stop the spread of waterborne diseases. The connected works still have problems with communication, mobility, accuracy, and scalability. A new SCADA system that incorporates IoT technologies was proposed in a real-time study for monitoring water quality. Using an Arduino Atmega 368 (Arduino Corporation, Somerville, MA, United States) and a Global System for Mobile Communication (GSM) module, it intends to detect water contamination, pipeline breaches, and automatic measurements of parameters (such as temperature sensor, flow sensor, and color sensor) in real time. The system is used in Tirunelveli, a significant city in Tamilnadu, India, to automatically capture sensor data from pressure, pH level, and energy sensors. The SCADA system now has more sensors at a lower cost. The outcomes demonstrate that the suggested approach performs better than those already in use and yields superior results. SCADA with GSM connectivity collects precise real-time sensor values for flow, temperature, color, and turbidity [30].

Safe water AI is one of the creative ways that AI is being used to achieve SDGs linked to water quality. Convolution Neural Network (CNN) and IoT technologies, developed in the USA, allow for real-time analysis and identification of pollutants like bacteria, even without an internet connection. The system is made out of cheap commercial off-the-shelf parts. The clean water AI package costs USD 500 right now. Further price reductions are anticipated as AI technology advances and is adopted by more individuals [31]. By 2050, 70% of the world's population is expected to live in cities. Uncontrolled urbanization can lead to cities that worsen poverty, inequality, informal settlements/communities, pollution, and unemployment. It can also encroach on bio-diverse areas and productive agricultural fields, releasing unchecked pollutants into vulnerable water supplies. Contrarily, multi-level governance, and integrated regional and urban planning can preserve and improve water resources, storage, and retention while encouraging investment in climate-resilient infrastructure, supporting stormwater management and disaster reduction, and boosting the blue economy [32].

Scientists and engineers can now reduce the inaccuracy associated with a system or particle's geometry or size by using AIs. The method that is most frequently used to accomplish this is to train an AI model using data that comes from systems whose behavior is already well understood. These methods are beneficial for nano-materials because it can frequently be challenging to reproduce the various effects and phenomena observed in materials like graphene. This program has a great deal of potential. In fact, it promises to incorporate machine learning into production methods, which would catalyze the advancement of both AI and nanotechnologies in the future [33]. There is an increased demand for fresh water in many crowded, growing cities worldwide, and planners are unsure how to meet this demand in the future. Communities may use such technology to breathe easily by extending the concept of affordable, clean, and sustainable water to the ecosystem as a whole. Understanding the primary environmental problems the world is currently facing and taking into account potential solutions from emerging nanotechnologies can help achieve sustainability in clean water [34]. There are now numerous ways to clean drinking water, including chemical processes that release toxins into the liquid media, killing cyanobacteria and causing cell lysis to reduce the microbial burden [35].

Water sector evolution due to AI 

The water sector is embracing AI, which powers machine learning-based intelligent operations that maximize resource consumption and operational budgets for businesses. 1. To bring in intelligent infrastructure solutions, water and wastewater operations will invest in technology over the next ten years; 2. By lowering energy costs, optimizing the use of chemicals for treatment, and facilitating proactive asset maintenance, AI will result in considerable operating expenses (OpEx) reductions in water and wastewater operations; 3. AI will forecast emergencies, learn from them faster, and identify trends that might point to an impending break event. As a result, notifications will get better over time; 4. AI will offer powerful decision-making intelligence to help operators make critical decisions without having to evaluate complex variables independently. AI empowers operators with intelligent recommendations and machine learning-driven decision-making, whether it's controlling the operation of pumps, calculating chemical dosages, or selecting whether to maintain assets; 5. AI will optimize pump runtimes to ensure that energy is only used when necessary to reduce energy consumption for water and wastewater operations; 6. AI will maintain clean water at an affordable cost for both public and private use. To ensure that effluence criteria are followed, and compliance fines are avoided, AI learns from the distinctive features of your site; 7. AI will simplify data integrity by processing this heterogeneous data to make it clear, valuable, safe, and the basis for high-fidelity recommendations; 8. AI will run genuinely intelligent water systems. Organizations can seek data-driven, innovative management of water systems due to the deployment of AI. As a result, water management will be dependable, long-lasting, and affordable [36]. Table 1 lists several uses of artificial intelligence (AI) in the management of water resources, each system having its unique advantages.

**Table 1:** AI-based models for water optimization

Model Name	Description	Benefits
Reservoir Operation Optimization [37]	AI models analyze historical data, rainfall patterns, and water demand to optimize reservoir operations, maximizing water storage and release timing.	Ensures efficient water storage and release, improves flood control, and optimizes hydropower generation.
Water Allocation Optimization [38]	AI algorithms consider water demand, availability, and environmental constraints to optimize water allocation schemes, minimizing conflicts and maximizing efficiency.	Promotes equitable water distribution, reduces user conflicts, and improves water-use efficiency.
Drought Forecasting and Mitigation [39]	AI-based models analyze rainfall patterns, soil moisture levels, and climate data to predict and mitigate drought events.	Enables early warning systems, improves drought preparedness, and facilitates proactive water management.
Integrated Water Resource Management [40]	AI-driven systems integrate data from multiple sources, including weather forecasts, river flows, and water usage, to provide holistic water resource management strategies.	It enhances decision-making processes, optimizes water allocation, and supports sustainable water management.
Environmental Impact Assessment [41]	AI models assess the environmental impact of water resource management activities, considering water quality, ecosystem health, and habitat preservation factors.	Facilitates environmentally sustainable practices, protects ecosystems, and preserves biodiversity.
Real-Time Water Monitoring and Control [42]	AI-powered sensors and data analytics enable real-time monitoring of water parameters and automatic control of water systems, optimizing water allocation and usage in response to changing conditions.	It improves operational efficiency, reduces water losses, and facilitates adaptive water management.
Stakeholder Engagement and Decision Support [43]	AI-based tools facilitate stakeholder engagement and provide decision support by analyzing data, generating insights, and fostering collaborative water resource management approaches.	Enhances communication and cooperation among stakeholders and supports evidence-based decision-making.

## Conclusions

To prevent microbiological contamination of drinking water sources or to reduce contamination to levels safe for human health, a variety of barriers are used from the start to when the water is used by the consumer. The precautions that promote safety include preserving and maintaining treated water quality, managing distribution systems (whether piped or otherwise), and protecting water resources. The best management strategy puts less emphasis on using treatment technologies to eliminate pathogens and more on preventing or minimizing their entrance into water sources. Rapid and sensitive identification of waterborne pathogens in drinking and recreational water sources is essential for the treatment and control of the spread of water-related diseases, especially in resource-constrained situations.

Localization is necessary before AI models, methods, and technology are implemented. The opportunities and problems associated with water differ by nation and place due to varying levels of infrastructure accessibility and implementation capabilities to address the issues and potential provided by AI. Before applying AI to address water-related problems, it is essential to conduct baseline studies to assess the implementation capabilities, return on investment, and impact of the intervention. Before implementing AI in the water sector, policymakers should thoroughly examine social, economic, and cultural issues. Support is required for AI for water-based activities to produce good development outcomes.

To ensure successful development outcomes, infrastructure development, and capacity-building policies should be linked with guidelines on using AI for water-related challenges. Skills development is necessary for all parties interested in water, and capacity development programs must consider this. These measures for increasing capacity should promote interdisciplinary and cross-disciplinary study and research. The basic requirements for computation, energy, the generation of data, and storage should be considered while developing infrastructure policies. It's crucial to arrange these policies correctly.

## References

[1] (2023). Drinking-water. https://www.who.int/news-room/fact-sheets/detail/drinking-water.

[2] Joy C, Sundar GN, Narmadha D (2023). Artificial intelligence based test systems to resist waterborne diseases by early and rapid identification of pathogens: a review. Sn Comput Sci.

[3] Melissa Denchak (2023). Water pollution: how to protect our source of life. https://www.iberdrola.com/sustainability/water-pollution.

[4] Cabral JP (2010). Water microbiology. Bacterial pathogens and water. Int J Environ Res Public Health.

[5] (2023). Interactive dialogue 2: Water for Sustainable Development. https://sdgs.un.org/sites/default/files/2023-01/23-00159%20Concept%20Paper%20UNWC_ID2_Website.pdf.

[6] Sinčak P, Ondo J, Kaposztasova D, Virčikova M, Vranayova Z, Sabol J (2014). Artificial intelligence in public health prevention of legionelosis in drinking water systems. Int J Environ Res Public Health.

[7] Nusrat F, Haque M, Rollend D, Christie G, Akanda AS (2022). A high-resolution earth observations and machine learning-based approach to forecast waterborne disease risk in post-disaster settings. Climate.

[8] Al-Sakkari EG, Abdeldayem OM, Genina EE, Amin L, Bahgat NT, Rene ER, El-Sherbiny IM (2020). New alginate-based interpenetrating polymer networks for water treatment: a response surface methodology based optimization study. Int J Biol Macromol.

[9] Calderón OAR, Abdeldayem OM, Pugazhendhi A, Rene ER (2020). Current updates and perspectives of biosorption technology: an alternative for the removal of heavy metals from wastewater. Curr Pollution Rep.

[10] Giri BS, Gun S, Pandey S (2020). Reusability of brilliant green dye contaminated wastewater using corncob biochar and Brevibacillus parabrevis: hybrid treatment and kinetic studies. Bioengineered.

[11] Cash RA, Halder SR, Husain M (2013). Reducing the health effect of natural hazards in Bangladesh. Lancet.

[12] Charnley GE, Kelman I, Gaythorpe KA, Murray KA (2021). Traits and risk factors of post-disaster infectious disease outbreaks: a systematic review. Sci Rep.

[13] Englande AJ Jr, Krenkel P, Shamas J (2015). Wastewater treatment & water reclamation. Reference Module in Earth Systems and Environmental Sciences.

[14] Tirivangasi HM (2018). Regional disaster risk management strategies for food security: probing Southern African development community channels for influencing national policy. Jamba.

[15] Reiner RC Jr, King AA, Emch M, Yunus M, Faruque AS, Pascual M (2012). Highly localized sensitivity to climate forcing drives endemic cholera in a megacity. Proc Natl Acad Sci U S A.

[16] Penrose K, de Castro MC, Werema J, Ryan ET (2010). Informal urban settlements and cholera risk in Dar es Salaam, Tanzania. PLoS Negl Trop Dis.

[17] Talavera A, Pérez EM (2009). Is cholera disease associated with poverty?. J Infect Dev Ctries.

[18] (2023). Satellites and cell phones form a cholera early-warning system. https://eos.org/science-updates/satellites-and-cell-phones-form-a-cholera-early-warning-system.

[19] (2023). Artificial Intelligence (AI): What it is and why it matters. https://www.sas.com/en_us/insights/analytics/what-is-artificial-intelligence.html.

[20] HC Info (2023). Artificial Intelligence: What It Is and How It Is Used. https://www.investopedia.com/terms/a/artificial-intelligence-ai.asp#:~:text=Artificial%20intelligence%20is%20based%20on,include%20mimicking%20human%20cognitive%20activity..

[21] Feyen Feyen, J.; Shannon, K.; Neville, M M (2023). What is Artificial Intelligence in 2023? Types, Trends, and Future of it?. https://www.mygreatlearning.com/blog/what-is-artificial-intelligence/.

[22] Sarker IH (2022). AI-based modeling: techniques, applications and research issues towards automation, intelligent and smart systems. SN Comput Sci.

[23] (2023). IBM builds a smarter planet. https://www.ibm.com/smarterplanet/us/en/.

[24] (2023). Top 5 World's Most Advanced AI Systems. https://www.pycodemates.com/2023/02/top-5-worlds-most-advanced-ai-systems.html.

[25] (2023). Where AI is Aiding Productivity. https://www.statista.com/chart/23779/ai-productivity-increase/.

[26] (2023). Definition of artificial intelligence. https://www.merriam-webster.com/dictionary/artificial%20intelligence.

[27] Jenny H, Wang Y, Garcia AE, Minguez R (2020). Using artificial intelligence for smart water management systems. ADB briefs.

[28] (2023). The Promise of Artificial Intelligence in Water Management. https://www.analyticsinsight.net/the-promise-of-artificial-intelligence-in-water-management/.

[29] Chhipi-Shrestha GK, Mian HR, Mohammadiun S, Rodriguez M, Hewage K, Sadiq R (2023). Digital water: artificial intelligence and soft computing applications for drinking water quality assessment. Clean Technol Environ Policy.

[30] Saravanan K, Anusuya E, Kumar R, Son LH (2018). Real-time water quality monitoring using Internet of Things in SCADA. Environ Monit Assess.

[31] Al-Adhaileh MH, Alsaade FW (2021). Modelling and prediction of water quality by using artificial intelligence. Sustainability.

[32] (2023). Concept Paper UNWC. https://sdgs.un.org/sites/default/files/2023-01/23-00159%20Concept%20Paper%20UNWC_ID2_Website.pdf.

[33] Post Post, Guest Guest (2023). AI and nanotechnology are working together to solve real-world problems - Stack Overflow. https://stackoverflow.blog/2022/03/21/ai-and-nanotechnology-are-working-together-to-solve-real-world-problems/.

[34] Nagar A, Pradeep T (2020). Clean water through nanotechnology: needs, gaps, and fulfillment. ACS Nano.

[35] (2023). Water and Sanitation. United Nations Sustainable Development. http://WaterandSanitation.UnitedNationsSustainableDevelopment.

[36] (2023). AI for water: 10 ways AI is changing the water industry. https://blogs.autodesk.com/innovyze/2022/05/01/ai-in-water-10-ways-ai-is-changing-the-water-industry/.

[37] Lai V, Huang YF, Koo CH, Ahmed AN, El-Shafie A (2022). A review of reservoir operation optimisations: from traditional models to metaheuristic algorithms. Arch Comput Methods Eng.

[38] Guo L, Xie X, Zeng J (2023). Optimization model of water resources allocation in coal mine area based on ecological environment priority. Water.

[39] Adikari KE, Shrestha S, Ratnayake DT, Budhathoki A, Mohanasundaram S, Dailey MN (2021). Evaluation of artificial intelligence models for flood and drought forecasting in arid and tropical regions. Environ Model Softw.

[40] Krishnan SR, Nallakaruppan MK, Chengoden R, Koppu S, Iyapparaja M, Sadhasivam J, Sethuraman S (2022). Smart water resource management using artificial intelligence—a review. Sustainability.

[41] Xiang X, Li Q, Khan S, Khalaf OI (2021). Urban water resource management for sustainable environment planning using artificial intelligence techniques. Environmental Impact Assessment Review.

[42] Chowdury MS, Emran TB, Ghosh S (2019). IoT based real-time river water quality monitoring system. Procedia Comput Sci.

[43] Maskrey SA, Mount NJ, Thorne CR, Dryden I (2016). Participatory modelling for stakeholder involvement in the development of flood risk management intervention options. Environ Model Softw.

